# Epidemiological trends and risk factors associated with dengue disease in Pakistan (1980–2014): a systematic literature search and analysis

**DOI:** 10.1186/s12889-018-5676-2

**Published:** 2018-06-15

**Authors:** Jehangir Khan, Inamullah Khan, Abdul Ghaffar, Bushra Khalid

**Affiliations:** 1 Department of Parasitology, Zhongshan School of Medicine, Sun Yat-sen University, Guangzhou, 510080 Guangdong China; 2 Sun Yat-sen University-Michigan State University Joint Center of Vector Control for Tropical Diseases, Zhongshan School of Medicine, Sun Yat-sen University, Guangzhou, 510080 Guangdong China; 3 Department of Zoology, Abdul Wali Khan University Mardan (AWKUM), Mardan, Khyber Pakhtunkhwa Pakistan; 4 Nuclear Institute for Food and Agriculture (NIFA), Peshawar, Khyber Pakhtunkhwa Pakistan; 5 Department of Meteorology, COMSATS University (CUI), Islamabad, Pakistan; 6 Evolution and Ecology Program, International Institute for Applied Systems Analysis, Laxenburg, Austria; 7 Earth System Physics, The Abdus Salam, International Centre for Theoretical Physics, Trieste, Italy; 8 Key Laboratory of Tropical Diseases and Control of the Ministry of Education, Guangzhou, 510080 China

## Abstract

**Background:**

Dengue is becoming more common in Pakistan with its alarming spreading rate. A historical review needs to be carried out to find the root causes of dengue dynamics, the factors responsible for its spread and lastly to formulate future strategies for its control.

**Methods:**

We searched (January, 2015) all the published literature between 1980 and 2014 to determine spread/burden of dengue disease in Pakistan.

**Results:**

A total of 81 reports were identified, showing high numbers of dengue cases in 2010, 2011, and 2013. The tendency of dengue to occur in younger than in older age groups was evident throughout the survey period and all four serotypes were recorded, with DENV1 the least common. Most dengue hemorrhagic fever (DHF) cases fell in the 20–45 years age range. High frequencies tended to be observed first in the Southern coastal region characterized by mild winters and humid warm summers and then the disease progressed towards the lowland areas of the Indus plain with cool winters, hot summers and monsoon rainfall. Based on this survey, new risk maps and infection estimates were identified reflecting public health burden imposed by dengue at the national level.

**Conclusions:**

Our study showed that dengue is common in the three provinces of Pakistan, i.e., Khyber Pakhtunkhwa (KP), Punjab and Sindh. Based on the literature review as well as on our study analysis the current expansion of dengue seems multifactorial and may include climate change, virus evolution, and societal factors such as rapid urbanization, population growth and development, socioeconomic factors, as well as global travel and trade. Due to inadequate remedial strategies, effective vector control measures are essential to target the dengue vector mosquito where high levels of human-vector contact occur. The known social, economic, and disease burden of dengue is alarming globally and it is evident that the wider impact of this disease is grossly underestimated. An international multi-sectoral response, outlined in the WHO Global Strategy for Dengue Prevention and Control, 2012–2020, is now essential to reduce the significant influence of this disease in Dengue endemic areas. Overall gaps were identified in knowledge around seroprevalence, dengue incidence, vector control, genotype evolution and age-stratified serotype circulation.

## Background

Dengue is a systemic mosquito-borne infectious disease caused by dengue virus (DENV) which has four common and genetically distinct serotypes [1, 2]. More than 50% of the human population lives in dengue endemic areas [3–6]. About 50 to 200 million dengue cases with 500,000 incidences of dengue hemorrhagic fever and over 20,000 deaths are being documented every year around the globe [7]. The annual economic burden of dengue is estimated at US$950 million and the disability-adjusted life year’s value is 372 (210–520) per million residents [8, 9].

Dengue is thought to be expanding in Pakistan. The first dengue case was reported from Hub, Baluchistan Province in 1960, when the estimated population of Pakistan was 45.9 million. The total number of reported dengue cases for the 1960–1980 period was only 12 [10–13]. The first serologically and virologically confirmed dengue outbreak was reported from Karachi in 1994 [10]. Since 1960, the population of Pakistan gone up to 188.2 million (2014), while the total number of reported dengue cases has increased up to 74,495, with 690 reported fatalities. The factors contributing to the nationwide spread of dengue virus and the increase in dengue incidence are poorly understood.

We have therefore, undertaken a comprehensive compilation of dengue cases from published data and known records in the country, and used a modeling framework to understand dengue prevalence and risk. We have considered socioeconomic, epidemiological and demographic factors, including sero-prevalence, serotype distribution and dengue vector transmission. In this review, we have also discussed possible routes of incursion of dengue to non-infected areas.

## Methods

Literature searching and analysis protocols followed the guidelines for “preferred reporting items of systematic reviews and meta-analyses” (PRISMA) [14]. Our searching strategy primarily was for dengue incidence and secondarily for dengue vectors. The protocol was registered on PROSPERO (managed by the Centre for Reviews and Dissemination, University of York) an internationally developed database of registered systematic reviews in health and social care. (http://www.crd.york.ac.uk/PROSPERO/display_record.asp?ID=CRD42015016696).

### Search strategy and selection criteria

As of January 2015, we searched several databases, such as Science Citation Index, Science Citation Index Expanded (SciSearch), Journal Citation Reports/Science Edition, Medline, SCOPUS, EMBASE, Google Scholar, CSA, ProQuest, CAB International, Biological Abstracts, BIOSIS, CAB Abstracts, CSA Environmental Sciences, Biology & Environmental Sciences, EBSCO Discovery Service, EM Biology, Global Health, PubMed, and Zoological Records. The published databases were accessed by using the terms dengue, dengue fever, climate change, dengue hemorrhagic fever (DHF), climate irregularities, risk factors and dengue fever, dengue fever and modeling, vector borne diseases, vector borne disease modeling, infectious disease surveillance early warning systems, and secondarily by choosing the terms dengue vectors, *Aedes aegypti* and *Aedes albopictus*, Pakistan. The search included studies published from January 1994 to December 2014 because dengue surveillance was established during this period.

#### Selection criteria

**Fig. 1:**
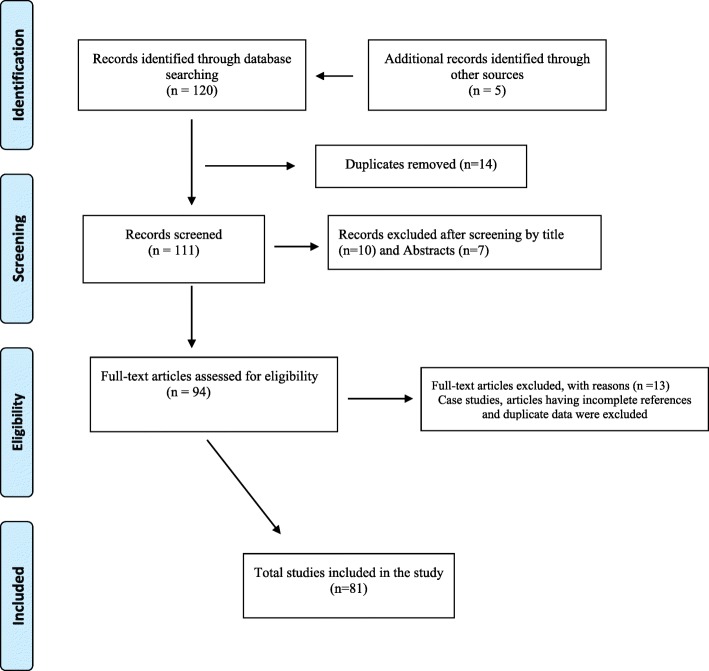
Results of literature search and evaluation of identified studies according to PRISMA

The articles were selected based on the following inclusion criteria: 1) peer-reviewed, 2) available full text articles, 3) published in English, and 4) only studies considering the distribution of dengue risk with increase in population along with socioeconomic, mosquito density, epidemiological and demographic factors, sero-prevalence, serotype distribution and dengue vector transmission, dengue case/infection or incidence. The rationale for inclusion criteria was to focus on the increase in population, mosquito density, and socioeconomic, ecological, epidemiological and demographic factors associated with dengue transmission. Articles not meeting the above criteria were excluded (Fig. 1).

#### Quality assessment and potential biases

Quality of each study was assessed through the combined criteria suggested by Effective Public Health Practice Project (EPHPP)1 and Wells et al. [15]. The quality of each study was determined across seven metrics: selection, study design, data collection, observational time period, interpretation of factors, and full description of dengue diagnosis. Potential bias within the studies was also determined.

**Fig. 2:**
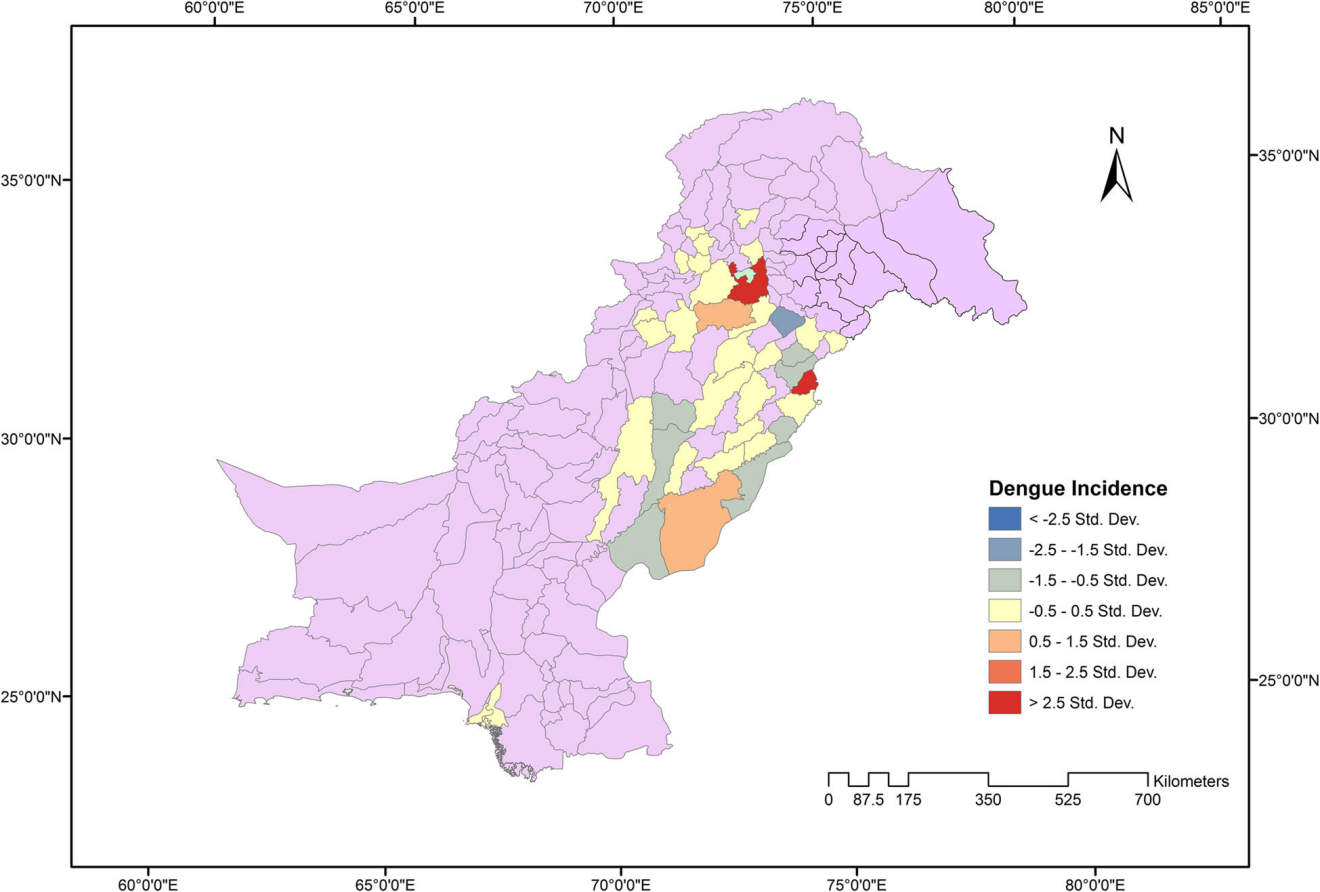
Relative risk ratio map (2011–2013) to model spatial variation in dengue incidences, population, and locality. Highly disease prone areas in dark red (Rawalpindi and Lahore), medium level disease prone areas in light red (Bahawalpur and Chakwal), highly vulnerable in yellow (Faisalabad, Karachi, Swat, Dera Ghazi Khan, Sargodha, Jhang, Hafizabad, Jhelum, Mianwali, Lakki Marwat, Bannu, Attock, Nowshehra, Peshawar, Mardan, Haripur, Narowal, Sialkot and Multan), medium level vulnerable areas in gray (Muzaffargarh, Layyah, Bahawalnagar, Rahimyar Khan, Sheikhupura and Gujranwala), and low level vulnerable areas in blue), whereas no record found in the scientific literature for purple colored areas

We mostly could not find human population data for respective areas where dengue cases (across a time course) were reported in the literature, and the literature we searched reported dengue cases rather than dengue incidence. This is a limitation of the current study. However, we developed a relative risk ratio map for 2011–2013, a period for which human population data was available [16–18]. Risk ratios were calculated with reference to the population and area of a region for 2011–2013 as shown in Fig. 2. The relative risk ratio was computed to model spatially varying relationships between dengue incidences, population, and locality.

## Results and discussion

A total of 81 out of 125 research reports were included in this review (Fig. 1). Dengue was found to be common in three provinces, Khyber Pakhtunkhwa (KP), Punjab and Sindh. There was some variation in disease burden which seems to be strongly influenced by vector presence, ecological factors like rainfall, temperature, travel and trade, socio-demographic factors and the degree of urbanization [16–20]. The following sections provide a detailed picture of factors influencing dengue incidence in Pakistan.

### Dengue vectors in Pakistan

*Ae. aegypti* was recorded for the first time by Barraud in 1934 [21] from Peshawar, Dera Ismail Khan, Lahore, Larkana and Karachi and thereafter from Lahore by Aslam Khan [22] (during 1969 and 1971). Qutubuddin [23] reported it from the Kohat-Hangu valley in 1949. After 1950, *Ae. aegypti* along with malarial mosquitoes seemed to have been suppressed [24] and until the 1980’s this species was not documented again [25]. In 1996 *Ae. aegypti* was reported in northern Pakistan from Landi Kotal near Peshawar in KP province [24] and from Karachi in 2010 [26, 27]. Both *Ae. aegypti* and *Ae. albopictus* were reported from Murree hills in the districts of Punjab [28], from Sindh by Mukhtar et al. [16] and from Charsadda, Nowshera, Peshawar, Mardan and Buner districts of KP in 2014–2015 by Khan et al. [29, 30]. It seems that both *Ae. aegypti* and *Ae. albopictus* have not only started re-invading formerly occupied areas (i. e. Karachi, Peshawar and Lahore) but have also started spreading to new areas where they were not present before (e.g. Attock, Haripur, Hasanabdal, Taxilla, Rawalpindi, Gujranwala, Sheikhupura, Faisalabad, Multan and Hyderabad) [5, 12, 31] (Table 1). *Ae. aegypti* and *Ae. albopictus* have been reported breeding in almost all types of artificial containers especially in tires [32] and other outdoor water storage containers [4]. Both species occur in different geographical areas of the country starting from sea level (Karachi) to higher altitudes (northern areas). In Southern parts of the country (24–50 m above sea level), only *Ae. aegypti* is prevalent, whereas in northern/sub mountainous areas (500–600 m above sea level with upper limit of 2500 m), *Ae. albopictus* seems dominant. In the central part of the country, both the species co-exist but *Ae. aegypti* appears dominant over *Ae. albopictus* [17].

**Table 1:** Dengue Vectors (mosquitoes) and their introduction to Pakistan

First Author	Year	Finding	Areas	Found in	Ref
Barraud	1934	Culicine fauna of British India was published	Peshawar, Dera Ismail Khan, Lahore, Larkana and Karachi in west Pakistan	1927–32	21
Qutubuddin	1960	First detection of *Ae. aegypti*	Kohat-Hangu valley	1949	23
Khan	1969, 1971	A check list on the names and taxonomic position of Culicidae in Pakistan was produced	West Pakistan	1960	22
Suleman	1996	Suppression of Malaria and *Ae. aegypti*	All cities that encountered with Malaria and Dengue in the past	After 1950’s	24
Kamimura	1986	Re-emergence of Malaria and Dengue	All cities that encountered with Malaria and Dengue previously	From 1980’s	25
Suleman Paul Tariq Tariq	1996 1998 2000 2010	*Ae. aegypti*	Landi Kotal, Karachi	1996–1997	24 26 27 32
Mukhtar	2011	Dominance of *Ae. albopictus* over *Ae. aegypti*	Northern/ sub mountainous areas (500–600 m above sea level)	2006–2010	19
Mukhtar	2011	Dominance of *Ae. aegypti* over *Ae. albopictus*	Central Pakistan region	2006–2010	19
Mukhtar Qasam Khan	2012 2014 2015	*Ae. aegypti* and *Ae. albopictus*	Muree hills, Charsadda, Nowshera, Peshawar, Mardan and Buner districts	2012, 2012–13,2014	18 28 29
Khan Khan Rasheed	2016 2017 2013	*Ae. aegypti* and *Ae. albopictus*	Karachi, Peshawar, Lahore, Attock, Haripur, Hasanabdal, Taxilla, Rawalpindi, Gujranwala, Swat, Buner, Sheikhupura, Faisalabad, Multan and Hyderabad	2013, 2014–15	5 31 13

### An overview of dengue in Pakistan

**Fig. 3:**
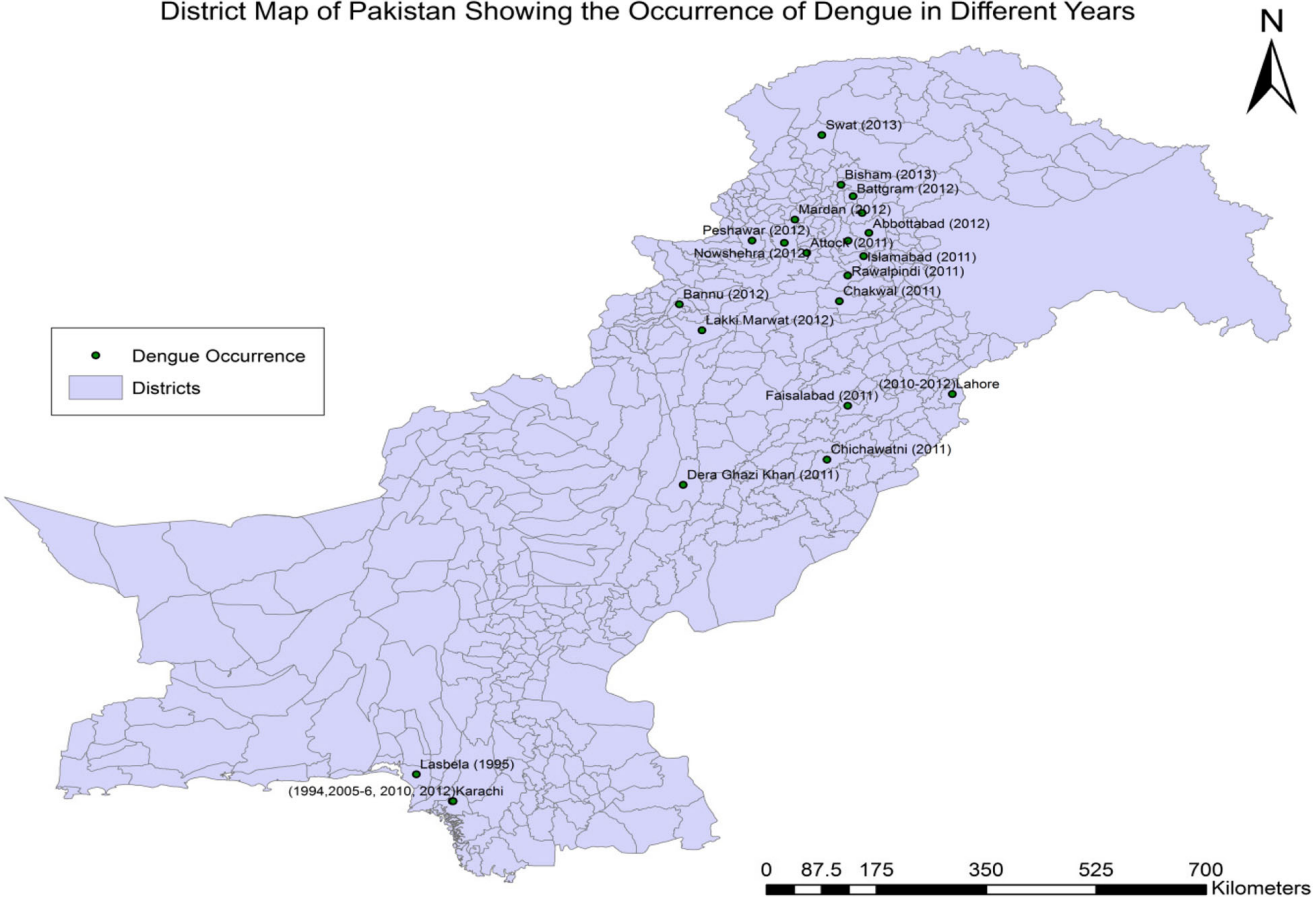
Spread of dengue disease in Pakistan with years indicated

Dengue serotype 2 was detected in 1987 and serotype 1 in 1990s [32]. The first outbreak was recorded in Karachi during 1994 caused by circulating DENV-1 and 2 [10, 11, 33–35]. Another dengue outbreak was documented in Karachi during 2005 in which DENV-1 and 2 were the predominant serotypes, with newly introduced DENV-3 also detected [33, 36, 37]. Three dengue virus serotypes (2, 3, and 4) were observed during 2008 in Lahore [33, 38, 39]. The largest dengue outbreak was reported during 2011 in Lahore with 22,562 dengue cases and 363 fatalities [1, 2, 5, 40]. The second largest epidemic occurred during 2013 in Swat (KP province) with 8343 dengue cases and 57 deaths (Fig. 3). The predominant circulating serotypes reported were DENV-2, 3 & 4 in Punjab during 2011, and DENV-2 & 3 in Swat, KP during 2013 [5, 39, 41] (Tables 2 and 3).

**Table 2:** The recorded dengue incidences and deaths in Pakistan (1982–2014)

Author	Year	No. of cases	Deaths	Found in	Reference
Idrees Rasheed	2012 2013	12	0	1982	34 12
Jamil	2007	145	1	1994	37
Idrees	2012	76	57	1995	34
Rasheed	2013	251	1	1997	12
Mukhtar	2011	1000	17	2003	19
Paul	1998	25	0	2004	32
Khanani	2011	3940	21	2005	70
Wasay	2008				54
Rasheed	2013	3000	52	2006	12
Khalid	2014	1208	22	2007	79
Ali	2013	2035	30	2008	58
Khan	2010	1099	16	2009	63
Humayun	2010	10,416	51	2010	39
		37,512	363	2011	
Khalid	2014	4833	2	2012	79
Khalid	2015a	8343	57	2013	16
Khalid Khan Khan Khan	2015b 2015a 2015b 2016	600	0	2014	17 4 29 5
**Total**		**74,495**	**690**

**Table 3:** The major dengue outbreaks documented in Pakistan

Outbreak	Year	District	Province	Serotypes	Incidences: Deaths
1st	1994	Karachi	Sindh	1 & 2	145:1
2nd	1995	Hub	Baluchistan	1 & 2	76: 57
3rd	2003	Haripur	KP	1 & 2	717:6
4th	2005	Karachi	Sindh	1, 2 & 3	3940:21
5th	2006	Karachi	Sindh	1,2 &3	3940:52
6th	2010	Karachi & Lahore	Sindh, Punjab	1, 2, 3 & 4	10,416:51
7th	2011	Lahore	Punjab	1, 2, 3 & 4	37,512:363
8th	2012	Karachi, Lahore & districts of KP	Sindh, Punjab, KP	2 & 3	4833:2
9th	2013	Swat and Besham	KP	2 & 3	8343:57

The highest numbers of confirmed dengue cases in Pakistan were noted during 2010 and 2011, with 37,512 and 10,416 cases respectively (Table 2). Even though dengue epidemics varied across the years, there was an increasing trend of dengue over time in Pakistan, suggesting a worsening situation since 2005 (Fig. 3). From 1994 to 2013, nine national epidemics (Table 3) occurred [5, 33, 39, 41, 42]. The reported dengue cases and fatality rates were very high in Lahore (2011) and Swat (2013) as compared to other Asian regions. However, the number of dengue cases reported in Pakistan is lower than other countries in Asia, South East Asia and Americas [43–45]. According to available climatic data [17], abnormal environmental conditions prevailed in the country during 2010, with heavy rains and flooding. Flood water accumulation in low lying areas (Lahore) of the country plus very high in-flow of people from surrounding cities for employment and education purposes [18] might be the reason for rise in dengue. Moreover, infested eggs of Aedes might have been carried with flood water and calm winds, resulting in dengue spread. Lahore, being the congested city coupled with lack of awareness and preparedness at the government level may have been the main reasons for high fatality rates during 2011. Additionally, Lahore borders India, and serotypes isolated from epidemics in Lahore show close resemblance with serotypes prevailing in India [43]; thus we cannot rule out the possibility of this serotype to be introduced. The introduction of the new serotypes DENV-3 (2005) and DENV-4 (2008) into Lahore likely increased the severity of disease in susceptible individuals.

The dengue outbreak of Swat occurred 2 years later than that of Lahore. The Swat region suffered from an unstable political environment and outflow of residents during 2008–9. Mud houses and tents were used by internally displaced people (IDPs) and many resided in open places close to standing water. The IDPs suffered from various enteric, digestive and intestinal diseases and were re-settled during 2010–11 but had lost their jobs. A severe dengue outbreak occurred 2 years post settlement among these IDPs. Khan et al. [4, 5, 7, 21, 30, 31] have reported transportation of old tires between Lahore, Karachi and Swat as the primary reason of this dengue outbreak in Swat, but severity of the disease was exacerbated by poor living conditions. Moreover, Swat is a mountainous area visited by people from all over the country especially Lahore, Rawalpindi, Faisalabad (Punjab) and Karachi (Sindh). This influx of visitors increased after the resettlement of IDPs in Swat and contributed to the introduction of virus in to Swat. The mild climatic condition of Swat provides suitable breeding conditions for Aedes [4, 5, 30, 31].

### Dengue epidemiology and ecological zones in Pakistan

**Fig. 4:**
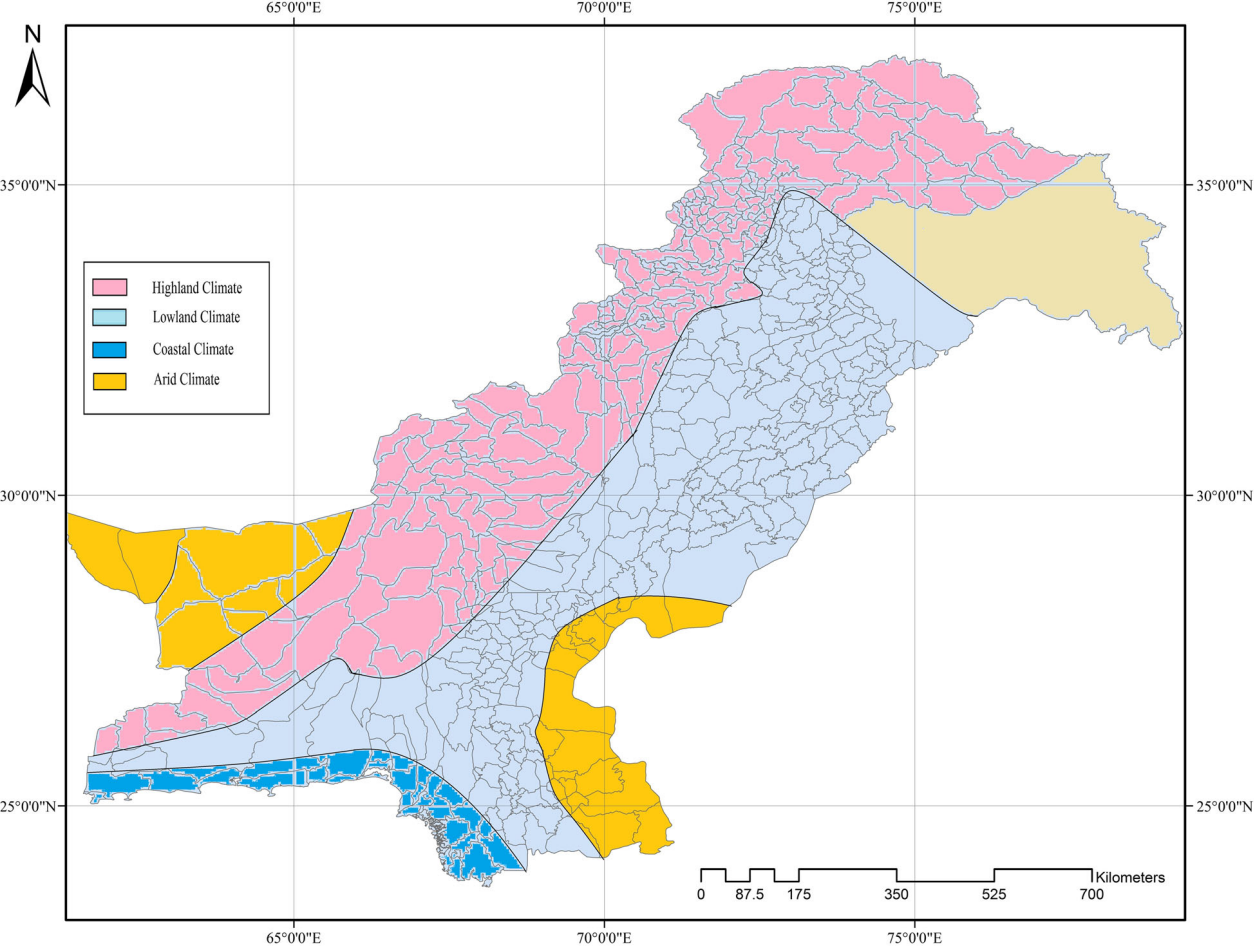
The different ecological zones of Pakistan

The geographic structure of Pakistan ranges from a mountainous northern part to a southern part with coastal plains that can be divided into four climatic regions: a highland climate, low land climate, costal climate and arid climate (Fig. 4). Zone A represents a hilly area, constituting the northern, north-west and the mountains of western region. Zone B represents the lower region (the Indus plain). Zone C encompasses coastal regions (Karachi coast, Makran coast and Indus Delta through the Rann of Kutch), while Zone D includes deserts of south eastern and south western Baluchistan having arid climate.

We collated regional (published) data from all the four provinces and climatic zones of Pakistan. Except one study [22], no published data was found for the south western region (Baluchistan). The reports indicate that dengue started in the coastal part of the country during 1980, moved to the lowland and finally to the highland climatic zones. The disease established in the coastal climate characterized by mild winter, humid and warm summers during the first 15 to 20 years (1980–2000) and then spread to the lowland areas of Indus plain characterized by cool winters, hot summers and monsoon rainfall. Karachi is one of the most populated cities in the coastal region of the country. It experienced the first dengue outbreak during 1994 [10]. Punjab and part of KP provinces are situated in the lowland Indus plain. During 2010–11, Punjab and especially Lahore were severely affected by dengue, followed by Faisalabad, Rawalpindi and some parts of KP [34, 35, 38, 46–50] and later on Swat in the north.

**Fig. 5:**
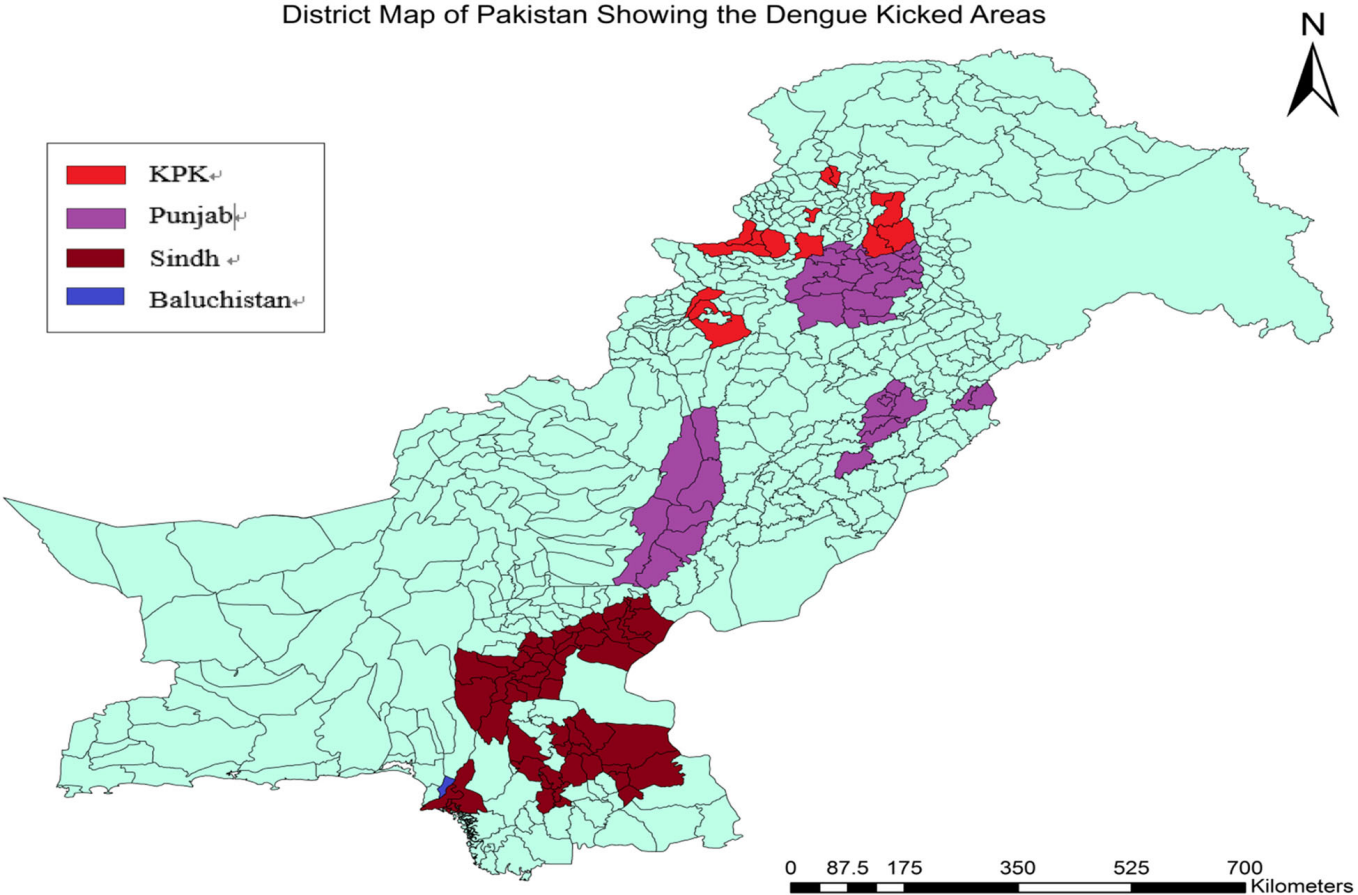
The dengue prone cities in different provinces of Pakistan are indicated in different colors

*Aedes* has extended its geographic distribution during the last 35-year review period, resulting in an increased number of dengue cases in all climatic zones of Pakistan. Figure 5 shows the distribution of dengue in different cities/provinces of Pakistan over the years since its first occurrence in the country. Initially in the survey, the coastal areas of Southeast (Karachi and Hub in Sindh and Baluchistan provinces) were highly affected by dengue, while from 2009 the highest number of cases were documented from the lowland Indus delta plain area (Lahore, Punjab province). Dengue cases reported in the southern coastal city (Karachi) were consistently lower than in other regions [51–55].

### Demographic patterns and dengue disease in Pakistan

The available data on dengue showed a change in age distribution over time (Table 4). Young adults (32–39 years) suffered highly from DF during 2003–2005 and 2011–2012 [1, 5, 34, 35, 51, 52]. However, in 1980s to 1994, DF was recorded in children aged < 16 years [34, 35, 47, 56, 57]. During 2006–2008, most DHF cases were recorded for 42–45 year age group, whereas in 2013, more cases of dengue were recorded in the 21–40 year age group [58–60]. In 2010, maximum dengue infections were recorded in individuals of more than 18 years. There is no age data available for 2008. However, in general individuals between 30 and 40 years age group showed an increase in hospitalization rates. More men than women (Table 4) were affected by dengue disease throughout Pakistan [48, 61–64]. This trend differs to the distribution of documented cases across sexes in countries in Latin America and other Asian countries [43, 65, 66].

**Table 4:** Distribution of dengue according to year, age and sex, and the relative abundance of serotypes

Author	Year	Main Findings			Found in	Reference
		RAS^a^ (%)	M:F^b^	Age years		
Khanani	2011	DENV-1, DENV-2(dominant)	N/A	< 16	1960s-1994	70
Paul	1998	N/A	95: 5	42	1995	32
Khan Koo Fatima Khanani Khaskheli Wasay Humayun	2010 2013 2011 2011 2010 2008 2010	N/A	63.2:36.8	32	2003	63 42 57 43 54 39
		DENV-2 (65), DENV-3 (34) DENV-1 (1)	67:33	20–40	2004–2005	
		DENV-2 (50), DENV-3 (50)	49:12	45	2006	
		DENV-2 (50), DENV-3 (50)	N/A	44.7	2007	
		DENV-2 (29), DENV-3 (12) DENV-4 (59)	N/A	N/A	2008	
Khanani	2011					70
Suleman	2017	DENV-2, DENV-3	27.1:39	21–30	2009	15
Khanani	2011					70
Usman	2011	DENV-2 (60), DENV-1(20)	60.4: 30.6	18–60	2010	77
Suleman	2017	DENV-3 (20)				15
Khan	2013	DEN-2 (91.03), DENV-2 & 3 (3.97), DENV-1(5)				75
Ahmed	2013					35
Ali	2013					58
Idrees	2012		80:20	21–40	2011	77
Mukhtar	2012					18
Koo	2013					42
Suleman	2017					15
Suleman	2017					80
Khan	2017	DENV-2 (77.7), DENV-3 (11.2)				31
Mukhtar	2012		73:26	34–45	2012	18
Suleman	2017					80
Khan	2015a	DENV-3 (66), DENV-2 (34)				4
Khan	2015b		68.6:31.3	20–40	2013	29
Suleman	2017					80
Khan	2016	DENV-2 (35), DENV-3 (65)				5
Suleman	2017a		67.3:32.7	20–45	2014	15
Suleman	2017b					80
Khan	2013					75
Ahmed	2013					35
Ali	2013					58
Idrees	2012					78
Mukhtar	2012					18
Khan	2013					41
Koo	2013					42

^a^*RAS* Relative abundance of serotypes ^b^M:F Male and Female ratio N/A: Not available/reported

### Distribution of virus serotypes

Until 1983, dengue cases were diagnosed by rapid test kits. The use of polymerase chain reaction (PCRs) for the isolation of dengue virus was limited. From 1994 onward, the Ministry of Health documented isolates of dengue virus in the country and DENV-1 & DENV-2 were observed. DENV-3 was reported in the 2005 epidemic and DENV-4 in 2008 (Table 4)**.** The reports also indicate a shift to DENV-2 & DENV-3 towards the end of the decade, with DENV-3 circulating as a dominant serotype in later epidemics. The data reveal similar patterns of serotype distribution to the national trends with some local variation [5, 29]. Most of the data was from the northeast part of the Sindh and Punjab Provinces [47, 51, 52, 67, 68] and the central-west region of KP [5, 7, 17, 18, 29, 56]. A parallel trend of serotype circulation was documented during 1960–2004 in the country (Tables 3 and 4). By 2012–13, nearly all dengue cases in the country were due to serotypes 2 & 3. In contrast, in the southeast region (including Lahore/Karachi), a prior shift in serotype may have happened with DENV-4 documented for 59% of dengue cases in 2008. However, for the period 1960–1994, DENV-2 was the predominant serotype responsible for the disease in the country. In the northern region of Pakistan, the outbreaks during 2003–2011 were mainly attributed to DENV-2 (> 50%) along with DENV-1 and DENV-3 [11, 33, 38]. During 2012, DENV-2 (77.8%) and DENV-3 (11.2%) were the major serotypes. Within the stated period, we observed an increase in the frequency of national epidemics [71] and severity of dengue disease (Figs. 3 & 5, Table 4). Some authors have attributed disease severity in children due to change in DENV-2 and DENV-3 versus DENV-1 and DENV-4 [5, 29, 33]. Nonetheless, the change in serotypes circulation may not be the only reason but other factors, such as regional variations in serotypes, viral strain virulence, serotype-specific immunity in individuals of different age, and the population density of *Aedes* mosquitoes may be involved. Several studies have documented clinical differences in dengue patients that could be linked with specific serotypes [2, 43, 76]. Patients infected by DENV-2 & DENV-3 showed greater severity (in terms of clinical differences, number of dengue cases and fatalities) in the disease than those infected with DENV-1 or DENV-4 [5, 62]. However, the appearance of DENV-3 in the country is linked to increased outbreaks, while the emergence of DENV-4 is not associated with increased disease. Secondary infection by other serotypes has been reported to be a significant factor for the increase in disease (dengue) severity [1, 2, 56, 57].

### Socio-demographic factors

An association of dengue disease with socio-economic, demographic and infrastructure features has been reported in many studies [5, 19, 20, 29]. Other reports have demonstrated increased risk factors for dengue disease in individuals living in single-storey homes with a higher density of residents per household [1, 5, 27, 69–71]. Studies have also documented that the emergence and distribution dynamics of DENV-3 and viral transmission intensity is associated with increased density of human population as well as the presence of susceptible individuals. Increased urbanization, poor sewerage infrastructure, and improper piped water supplies have increased the risk of dengue disease in the residents [5, 72]. Figure 5 shows the dengue prone cities in different provinces of Pakistan, while the district-wise severity of dengue incidence with respect to their population and area has been shown in Fig. 2. Some research reports have identified that low income families with large number of children and women are more exposed to dengue than those with higher socio-economic status [19, 20]. A report of sero-epidemiological study of 8000 randomly selected individuals during June–October (2013) in the same town confirmed that low income was an important factor for higher prevalence of dengue [4, 29]. The review indicates dissimilar patterns of dengue with time and space that suggests the involvement of diverse risk factors in transmission of the disease. Nonetheless, it is possible that the improper use of land, refugee influx, deforestation and unplanned urbanization also play a major role in dengue spread [17, 19].

### Genetic evolution and disease severity

The increase in geographic expansion of dengue over time has been associated with the emergence of endemic and epidemic (genetically diversified) genotypes due to small evolutionary changes in the gene pool of serotypes [2, 72]. The introduction of a new clade due to genetic changes in an area results in dengue epidemics (DHF and DSS) [33, 73]. Variation in serotypes/genotypes could reflect genetic bottlenecks and/or natural selection [71, 75]. In Pakistan, heterogeneity in disease may be, at least in part, due to the repeated introduction of new strains of DENV from other parts of the world, resulting in a conversion from hypoendemicity to hyperendemicity [4, 29].

Serotype 1 has been circulating in Pakistan since before the 1990s, with two genotypes (I and IV). Genotype I is closely related to DENV-1 that was responsible for 2009–2010 dengue outbreaks in Sri Lanka [72]. These strains have also been reported from Saudi Arabia, Thailand and Malaysia. Serotype 2 is the major circulating serotype in Karachi, Lahore, Faisalabad and Rawalpindi since 1994 [11]. There are repeated extinctions of serotype 2 and 3,which are replaced by new variants in the country [72]. DENV-2 has genotype IV (major) which was introduced in Pakistan from India in the late 1980s and from Sri Lanka around 2000 [2, 11]. This strain has also genetic resemblance with the Chinese (circulated during 1999 and 2000) and Saudi Arabian strains [41]. DENV-3 was first reported in 2005 dengue outbreak in Karachi, Pakistan. Serotype 3 isolated during the 2005 and 2006 outbreaks belong to genotype III and is most closely related to serotypes reported from India [36] and Sri Lanka [11, 72]. Furthermore, all detected serotypes during 2006 to 2009 in Karachi and Hyderabad (Southern Pakistan) share genetic similarity with two Indian and one Chinese strains that circulated in the Indian subcontinent prior to their emergence in Pakistan during 2005–2006 [41]. Similarly, serotype 4 first detected during the 2008 dengue outbreak in Lahore, Pakistan [38] has highest resemblance with the strains detected in 2007 in Andra Pradesh (South India). Human (dengue infected) migration, vector dispersion and virus evolution are critical in the recent geographic increase of dengue to areas where the disease was not a major public health threat previously.

### Factors leading to disease spread in Pakistan

#### Climate

The growth and propagation of *Aedes* is dependent on climate and can be linked to dengue incidence in a region [5, 17, 18]. The gonotropic cycle of vector mosquito and the extrinsic incubation period of dengue virus are inversely related to high temperature, which increases egg laying activity and blood meal frequency, with increased risk of viral transmission [32]. A seasonal pattern of dengue linked to climate, occurs in different cities of Pakistan [5, 16, 17, 19, 20]. The highest dengue cases occur during July–September with more rainfall, optimum temperature and humidity, providing a conducive environment for breeding, survival and growth of *Aedes* mosquitoes [5]. It has been hypothesized that increases in the average temperature and humidity around the globe will increase the potential of dengue epidemic in approximately 50–60% of the global population by 2085 [75].

#### Globalization, travel, and trade factors

A change in climate may not be the only factor affecting dengue spread, but travel and trade may also be important. Increases in travel during the twentieth century have resulted in a 40-fold increase in dengue [34, 70]. Overcrowded airports provide ideal mosquito breeding spots and DENV distribution points when people travel to different countries [75]. The range of adaptations of *Aedes* mosquitoes for breeding and surviving within trapped water in old tires and other goods has increased the risk of dengue in the past few decades; and the situation may be aggravated as more automobiles are used globally [41, 64, 71].

### Socioeconomic factors

Socioeconomic factors such as population growth, improper urbanization, settlement and other socioeconomic constraints on control measures contributes to the recent spread of dengue [71]. The construction of necessary infrastructures for water collection, storage, and disposal in urban and peri-urban settlements has provided favorable ecological niches and breeding sites for dengue vectors. The increase in population density of mosquito as well as humans, as part of urban population growth, has increased the vectoral capacity of vector mosquitoes and hence the transmission of dengue [29, 30]. A study by Khalid and Ghaffar [17] conducted in two cities, Rawalpindi and Islamabad, found that urbanization, hydrological conditions (stream flow and stream density), House Density Index (HDI) including house type, urbanization types, indoor and outdoor conditions overall form a complex structure to promote dengue occurrence and transmission in these two areas. The energy crises in Pakistan have aggravated the situation further where the low income families are compelled to sleep in the open environment and thus are exposed to mosquitoes. The government is struggling to overcome this situation and has increased its spending on construction of hydropowered and nuclear powered stations for generating electricity [48, 69–82].

### Limitations of the study

While this review has attempted to be comprehensive, several limitations exist around existing studies. No data was available on the seroprevalence of virus prior to 2010. Similarly, human population data and the number of dengue case were often unclear over the study period, and dengue cases rather than incidences were reported.

## Conclusion

Currently more than 125 countries including Pakistan are dengue endemic. This review showed dengue to be common in three provinces of Pakistan, i.e., KP, Punjab and Sindh. The expansion of dengue in Pakistan is likely to be due to multiple factors which may include a change in climate, evolution of virus, and social factors like increased urbanization, higher population growth and development, socioeconomic factors, and worldwide travel and trade. More effective control measures for dengue mosquitoes are necessary in locations where humans interact with vector species. Globally, dengue is expanding to non-endemic areas. The Worldwide Strategy for Prevention and Control of Dengue as outlined by WHO (2012–2020), needs to be implemented to limit the spread and impact of this disease. Recommended control measures for dengue may include population (vector) suppression using the ecofriendly control techniques, utilization of air conditioning, window/door screening in homes and offices, improved water storage practices and waste material disposal infrastructure, which can reduce the breeding sites of dengue vectors.

## Abbreviations

DHF: Dengue Hemorrhagic Fever
HDI: House Density Index
IDPs: Internally Displaced Peoples
KP: Khyber Pakhtunkhwa
